# Exploring the Costs of Hospital and Emergency Department Utilisation in the First Three Years After Diagnosis for Adults Diagnosed With Pancreatic Cancer in Queensland, Australia

**DOI:** 10.1002/cam4.71193

**Published:** 2025-09-04

**Authors:** Shafkat Jahan, Daniel Lindsay, Abbey Diaz, Ming Li, Joan Cunningham, Gail Garvey

**Affiliations:** ^1^ First Nations Cancer and Wellbeing Research Program, Faculty of Health, Medicine and Behavioural Sciences, School of Public Health The University of Queensland Brisbane Queensland Australia; ^2^ Cancer Health Economics Cancer Council Queensland Brisbane Queensland Australia; ^3^ The National Centre for Aboriginal and Torres Strait Islander Wellbeing Research The Australian National University Canberra Australian Capital Territory Australia; ^4^ Menzies School of Health Research Charles Darwin University Darwin Northern Territory Australia

**Keywords:** cancer treatment, health care cost, hospital episodes, pancreatic cancer

## Abstract

**Objective:**

To quantify costs incurred by the health system for hospital episodes and emergency department (ED) presentations for pancreatic cancer patients within the first three years after diagnosis in Queensland, Australia.

**Study Settings and Design:**

Using a linked administrative dataset, CancerCostMod, which includes cancer diagnoses from the Queensland Cancer Registry (1st July 2011–30th June 2015) and linked Queensland Health Admitted Patient Data Collection and ED Information Systems records (1st July 2011–30th June 2018), we assessed costs for adults diagnosed with primary pancreatic cancer (International Classification of Diseases, 10th Revision: C25). Costs (in Australian dollars) were assigned using national public costs and private hospital charge datasets for the relevant year. Descriptive analyses were conducted to evaluate hospital and ED utilization and costs. Cost variations across sociodemographic and clinical characteristics were assessed using Kruskal–Wallis or Mann–Whitney *U* tests.

**Principal Findings:**

Among 2082 individuals diagnosed with pancreatic cancer, hospital episodes (*n* = 26,405) in the first three years after diagnosis cost a total of $100.7 million; median cost per patient was $36,832. For ED presentations (*n* = 4228), corresponding figures were $3.6 million (total) and $963 (median per patient). Most of the total hospital (81%) and ED (79%) costs occurred in the first year after diagnosis. Patients who survived ≤ 6 months had the lowest median cost per patient but accounted for 38% of total hospital costs. Median cost per patient varied substantially by socio‐demographic (i.e., Age groups, Indigenous status, socio‐economic disadvantages) and clinical characteristics (i.e., comorbidity, cancer morphology, location of tumor, tumor resection, palliative care).

**Conclusions:**

Our findings highlight the significant economic burden of pancreatic cancer on the healthcare system, especially within the first year. Targeted strategies are essential to optimize healthcare delivery, ensure equitable access, and improve outcomes.

## 1 Introduction

Pancreatic cancer was the eighth most commonly diagnosed type of cancer and the fourth leading cause of cancer death in Australia in 2023 [1, 2]. Despite its poor prognosis and short survival, pancreatic cancer diagnoses impose a significant economic burden on healthcare systems [3, 4]. In Australia, healthcare expenditure related to pancreatic cancer was approximately $60 million Australian dollars (AUD) in 2015 to 2016, predominantly attributed to hospital admissions [5].

At the national level, inpatient care is the largest component of cancer‐related healthcare spending in Australia, accounting for 58% of total costs (36% for public and 22% for private hospital admissions) [5]. Although there is a gradual shift toward outpatient delivery of some oncology services, inpatient care remains the most resource‐intensive component, contributing substantially to the overall financial burden of cancer treatment in Australia.

A systematic review of European studies reported considerable variability in healthcare costs for pancreatic cancer, ranging from EUR €802 to EUR €353,099, with an average cost of EUR €40,357 per patient [3]. An Australian study ranked pancreatic cancer third among cancers in terms of annual mean excess costs compared to cancer‐free controls [4]. A high proportion of costs occurred immediately after diagnosis and at the end of life, with hospital‐based costs accounting for 70% of the total costs immediately post‐diagnosis and 84% of the total costs at the end of life. However, the small sample size (*n* = 157) may limit the accuracy of these estimates, and cost estimates may vary across studies due to differences in methodological approaches.

Health service utilisation and related costs for pancreatic cancer patients are influenced by various factors, including patient and disease characteristics (e.g., age, tumour type, stage of disease, remoteness, and socioeconomic status), as well as the type of care received (e.g., surgical, chemotherapy, or palliative care) [6, 7, 8, 9, 10, 11, 12]. While some studies have highlighted higher hospitalisation costs for younger patients [6, 10], the impact of sociodemographic disparities on healthcare costs remains underexplored. Understanding these variations is critical for informing equitable healthcare delivery and optimising resource allocation.

The recently launched Australian Cancer Plan [13] aims to improve health equity for First Nations Australians and people living in rural and remote areas, strongly emphasizing equity in cancer care services. To achieve this, it is necessary to identify and address existing disparities across different population groups in cancer outcomes and access to services.

Limited research has explored the distribution of costs associated with pancreatic cancer across the continuum of cancer care in Australia, a country with a universal health care system complemented by optional private health insurance. Linked administrative datasets allow a detailed exploration of costs to the Australian healthcare system.

This study used linked administrative data to quantify costs incurred by the healthcare system for hospital episodes and emergency department (ED) presentations for individuals following a pancreatic cancer diagnosis in Queensland, Australia between July 2011 and June 2015. The analysis focuses solely on healthcare system costs, excluding out‐of‐pocket expenses. By examining these costs across various patient and treatment characteristics, this study seeks to provide critical insights to help policymakers and healthcare providers optimize resource allocation and improve equity in cancer care. These findings also offer valuable implications for global strategies aimed at reducing disparities in healthcare access and outcomes across different healthcare systems.

## 2 Methods

### 2.1 Study Design and Study Population

The population for this study was nested in a larger dataset, CancerCostMod [14], the development of which is described elsewhere [15]. Initially, CancerCostMod used cancer cohort data from 1 July 2011 to 30 June 2012. To improve sample size and capture longer‐term outcomes, the date range was extended to 30 June 2015, with health record linkage expanded to 30 June 2018, ensuring at least three years of follow‐up data. Briefly, the base population for CancerCostMod comprised all cancer diagnoses (excluding keratinocyte cancers) in Queensland between 1 July 2011 and 30 June 2015, as recorded by the Queensland Cancer Registry (QCR) (*N* = 106,571 patients). Each individual's QCR record was linked to their records in the Queensland Health Admitted Patient Data Collection (QHAPDC) and Queensland Health Emergency Department Information Systems (EDIS) from the date of diagnosis onward, covering the period from 1 July 2011 to 30 June 2018. Only records on or after the date of diagnosis were included, with follow‐up data censored at the time of death or three years post‐diagnosis, whichever occurred first.

The analysis focused on records from the study period where pancreatic cancer (ICDO‐C25) was reported as the primary cancer diagnosis in individuals aged 18 years or older at the time of diagnosis (*N* = 2082). Only malignant pancreatic cancers were included, identified using ICD–O‐3 behaviour code/3 [16]. Pancreatic neuroendocrine tumors (PNETs) (ICD code 25.4) were excluded due to their distinct origin, prognosis, and survival rates [17]. However, this exclusion has minimal impact on total cost estimates due to the limited number of cases (*n* = <six).

Morphology codes were classified using the WHO Classification of Tumours of the Digestive System, 5th edition [18]. Cases were grouped into: (1) pancreatic ductal adenocarcinoma (PDAC); (2) non‐PDAC pancreatic malignancies; and (3) pancreatic neuroendocrine neoplasms (PNENs). Cases with non‐specific morphology codes such as 8000/3 (Neoplasm NOS) and 8010/3 (Carcinoma NOS) were retained for total cost estimates to avoid underestimation but were not included in group comparisons due to limited pathological specificity. A full list of morphology groupings is provided in Table S1.

The QCR database includes sociodemographic characteristics at the time of diagnosis, such as age, sex, Indigenous status, and residential postcode. Patient postcodes were mapped to the Index of Relative Socio–Economic Disadvantage (IRSD), a summary of the economic and social conditions of an area. The IRSD was collapsed into quintiles (Q) ranging from Q1 (most disadvantaged) to Q5 (least disadvantaged) [19]. Postcodes were also mapped to the Australian Statistical Geography Standard (ASGS) Remoteness Structure, which classifies areas based on distance from services and population size, into metropolitan, regional (inner and outer), or remote (remote and very remote) [20]. Individuals with missing postcode (*n* = 16) were excluded from analyses of variations in average hospital episodes, ED presentations, and associated costs by remoteness and IRSD.

Palliative care episodes were identified using the ‘care type’ variable within the QHAPDC dataset. ICD codes reported in the QHAPDC dataset were mapped to the Charlson Comorbidity Index (CCI) to create a weighted score for patient comorbidities [21]. Scores relating to ‘any malignancy, including lymphoma and leukemia, except malignant neoplasm of skin,’ and ‘metastatic solid tumor’ were excluded from the CCI score calculation, as cancer was assumed to be the primary diagnosis in this dataset. Patients undergoing pancreatic surgery were identified through the relevant Australian Classification of Health Interventions (ACHI) procedure code (see Table S2 for a list) [22]. The morphology of the cancer and the cancer site were also determined using ICD codes (ICD–O morphology code and site codes).

### 2.2 Assigning Costs for Hospital Episodes and Emergency Department Presentations

In Australia's universal healthcare system, hospital funding comes from a mixture of the Australian Government, State/Territory Government, individual out‐of‐pocket payments, Department of Veteran's Affairs (DVA), private health insurance, and other sources [23]. Medicare funds provide free or subsidized primary healthcare services outside of hospitals. Public hospital services are primarily funded by State and Territory Governments, while private health insurance is optional. In 2016–2017, 92% of public hospital funding was from the Australian (41%) and State (51%) Governments, while private hospitals received 31% of their funding from government sources and 69% from non‐government sources [24].

The QHAPDC and EDIS datasets contain all hospital episodes and ED presentations at Queensland's public and private hospitals. For public hospital episodes, costs were assigned based on the Australian Refined Diagnosis‐Related Group (AR‐DRG) classification, using the average estimated cost per episode reported by the National Hospital Cost Data Collection (NHCDC) for the relevant year [25]. To account for possible variations in healthcare delivery costs across different patient demographics, prior to analyses, we adjusted the expenses for each activity according to certain patient characteristics (e.g., First Nations Australians and those living in more remote areas) using estimates produced by the Individual Hospital Pricing Authority (IHPA) [26]. These IHPA adjustments reflect higher resource use and complexity of care in these populations, thereby improving the accuracy and relevance of cost estimates for these groups. However, no adjustments were made for the private hospital episodes and ED presentation costs, as there was no equivalent to the IHPA's adjustments for these services.

For private hospital separations, we assigned an estimated charge per separation using the average charge per separation for the relevant AR‐DRG, as published in the Private Hospital Data Bureau (PHDB) Annual Reports [27]. These represent the billed charges, not the actual cost of delivering care.

The use of estimated costs for public hospitals and estimated charges for private hospitals, with adjustments only available for the public sector, introduces heterogeneity in the data. These differences should be considered when interpreting comparative analyses across hospital types. Also, our cost estimates are based solely on AR‐DRG assigned costs (public) and average charges per AR‐DRG (private) and therefore do not capture costs incurred outside the DRG system.

Each ED presentation was coded to the ED classification system Urgency‐Related Group (URG) using the triage category, discharge destination, and the primary reason for attending the ED (ICD–10–AM). The cost attributed to each URG for each ED presentation was assigned using the mean cost per presentation reported by the NHCDC Report for the relevant year [24].

To quantify healthcare utilization and associated costs post‐diagnosis, data were limited to all hospital episodes and ED presentations within 36 months after the date of diagnosis (provided by QCR).

We analysed hospital episodes and ED presentations separately. Hospital episodes and ED presentations costs were calculated for each month from the date of diagnosis (t = 0) to 36 months post–diagnosis or until the person died, whichever came first. Only the month and year of diagnosis were available for our analysis, as this was a condition of the data release. Total costs represent the aggregated healthcare expenses over the 36‐month period following pancreatic cancer diagnosis. Average costs reflect the per‐person costs to the health system for hospital episodes (inpatient admissions and ED presentations), excluding non‐hospital care (e.g., primary care, specialists' visits outside hospital) over 36 months post–diagnosis, or for every month they were alive within that 36‐month period. If an individual had no health services for the month, the cost was recorded as AUD $0. If an individual died during the study period, costs were not recorded for the months following death. All costs are reported in AUD adjusted to the 2020 calendar year [28].

### 2.3 Statistical Analysis

We conducted descriptive analyses to identify the demographic characteristics and the number of hospital episodes and ED presentations to the Queensland healthcare system over the 36‐month period after pancreatic cancer diagnosis, along with total and median costs per person. To account for the non‐normal distribution of the data, median costs and episodes were reported. Monthly costs were calculated for each individual. As the dataset only provided the month and year of service, the exact timing of specific treatments or episodes of care could not be determined. These monthly costs were then aggregated into three distinct time periods: 0–12 months, 13–24 months, and 25–36 months. For each period, the total hospital episodes and ED presentations and associated costs were summed, including both individuals who were alive and those who had died in order to provide a comprehensive assessment of health service use and associated costs across the entire sample. However, for calculating the median number of hospital episodes and ED presentations and associated costs, we included only individuals who were alive at the start of each time period (i.e., at 0 months, 13 months and 25 months). This approach ensured that the median accurately reflected the healthcare use and costs for those who had the opportunity to use services each time period, without being skewed by those who were deceased. This distinction clarifies that the median costs are conditional on survival at the start of each period, while total costs are reported for the entire cohort.

As first‐year hospital episodes, ED presentations, and associated costs were significantly higher than in subsequent years, we further calculated the median cost per person during the first year conditional on survival length, categorizing individuals into five mutually exclusive groups based on their total survival following diagnosis: 0 to 6 months, 7 to 12 months, 13 to 24 months, 25 to 36 months, and > 36 months.

To analyse cost variations by length of stay (LOS), we calculated the total LOS per person by summing the LOS across all episodes for each individual and categorised it into three groups: ≤ 7 days, 8 to ≤ 28 days, and > 28 days (as 39% of individuals had a total LOS > 4 weeks). For each LOS group, we calculated total costs, total hospital episodes, and median costs and episodes (Table S3). These analyses allowed us to identify service use and cost patterns associated with prolonged hospitalisation.

We also explored median costs per person during the first year following diagnosis, based on different socio‐demographic characteristics, such as age group, sex, Indigenous status, remoteness, socioeconomic disadvantage of their place of residence, comorbidity score, location of the tumor within the pancreas, whether or not resection of the primary tumor occurred, and hospital‐based palliative care. Mann–Whitney *U* tests or Kruskal–Wallis tests were employed to compare differences in costs and episodes across these characteristics (*p* value < 0.05). We focused on this timeframe due to the notably higher median costs, hospital episodes, and ED presentations during the first year. We also examined cost variations across these groups based on whether they underwent tumor resection, given the significantly higher costs associated with tumor resection. We also explored health service use and costs for the first 6 months and 3 years post‐diagnosis across different characteristics to assess the differences in health service use patterns and costs between months and 2 months and 36 months post‐diagnosis (Tables S4 and S5). All analyses were performed using SAS V9.4 [29].

## 3 Results

Between 1 July 2011 and 30 June 2015, 2082 adults aged 18 years and over were diagnosed with pancreatic cancer in Queensland. Table 1 presents their demographic characteristics at the time of diagnosis. The mean age at diagnosis was 70.7 years (SD: 12.5), and a Kaplan–Meier analysis shows that the median survival time was 5 months (IQR = 2–12), which did not change substantially over the study period.

**TABLE 1:** Demographic characteristics of Queensland adults diagnosed with pancreatic cancer between 1 July 2011 and 30 June 2015 (*N* = 2082).

Characteristics	*n* (%)
Age group (years)	
18–50	137 (7)
51–65	511 (24)
66–75	657 (32)
76+	777 (37)
Sex	
Male	1103 (53)
Female	979 (47)
Survival time	
≤ 6 months	1039 (50)
7–12 months	374 (18)
13–24 months	280 (13)
25–36 months	107 (5)
> 36 months	282 (14)
Indigenous status	
First nations	48 (2)
Other Australians	2034 (98)
Remoteness area^a^	
Metropolitan	987 (48)
Regional	933 (45)
Remote	146 (7)
Index of relative socio–economic disadvantage^a^	
Quintile 1 (most disadvantaged)	181 (9)
Quintile 2	85 (4)
Quintile 3	361 (17)
Quintile 4	940 (45)
Quintile 5 (least disadvantaged)	499 (24)
Morphology	
Adenocarcinoma	1309 (63)
Carcinoma	43 (2)
Neuroendocrine carcinoma	155 (8)
Neoplasm and unspecified	575 (27)
Charlson Comorbidity Index^b^	
0	1266 (61)
1	233 (11)
2 or more	583 (28)
Cancer site	
Head of pancreas	926 (44)
Tail of pancreas	255 (12)
Body of pancreas	200 (10)
Other sites of pancreas^c^	191 (10)
Not specified	510 (24)
Palliative care episode^d^	
Yes	969 (46)
No	1113 (54)
Underwent tumour resection	
Yes	379 (18)
No	1703 (82)

^a^ Excludes those with missing postcode (*n* = 16).

^b^ Charlson Comorbidity Index: score calculation excludes cancers (e.g., malignancies and metastatic tumors), as cancer is considered the primary diagnosis in this dataset.

^c^ Other sites of the pancreas include pancreatic duct (*n* = 21), neck of pancreas (*n* = 90), and overlapping lesion of pancreas (*n* = 80).

^d^ At least one palliative care episode is recorded.

### 3.1 Hospital and ED Use and Associated Costs in the First 3 Years After Diagnosis

There were 26,405 hospital episodes during the first three years following a pancreatic cancer diagnosis, costing the Queensland healthcare system a total of $100.7 million. Most people with pancreatic cancer (68%) died within 1 year of diagnosis. Most of the total hospital costs (81%) and episodes (76%) also occurred within this period (see Table 2).

**TABLE 2:** Costs (in $AUD) and numbers of hospital episodes and ED presentations within the first three years after diagnosis for Queensland adults diagnosed with pancreatic cancer between 1 July 2011 and 30 June 2015.

Months since diagnosis	0–6 (*n* = 2082)^a^	7–12 (*n* = 1043)^a^	13–24 (*n* = 669)^a^	25–36 (*n* = 389)^a^	Total (*n* = 2082)
Hospital episodes					
Median cost per person (IQR)	$24,015	$44,412 (26,807–70,680)	$16,153	$17,298	$36,832 (19,846–62,898)
	(13,528–41,044)		(4447–34,397)	(5150–38,234)	
Total cost ($)	$62,218,612	$19,689,122	$12,265,298	$6,487,806	$100,660,838
Median episodes per person (IQR)	4 (2–10)	10 (5–21)	4 (1–11)	4 (1–13)	6 (3–15)
Total episodes, *n*	14,880	5061	4247	2217	26,405
ED presentations					
Median cost per person (IQR)	$550	$1035	$0	$210	$963
	(0–1585)	(0–2886)	(0–971)	(0–1793)	(0–2293)
Total cost ($)	$2,079,178	$702,320	$479,413	$279,566	$3,557,807
Median episodes per person (IQR)	1 (0–2)	1 (0–3)	0 (0–1)	1 (0–2)	1 (0–3)
Total episodes, *n*	2494	831	581	322	4228

^a^ Number of people alive at the beginning of each time point.

There were 4228 ED presentations in the first three years following pancreatic cancer diagnosis; the total cost to the Queensland healthcare system was $3.6 million. The median cost per person for ED presentations following their diagnosis was $963 (IQR $0–$2293). Most costs to the healthcare system for ED presentations (79%) occurred during the first 12 months following a pancreatic cancer diagnosis, with an estimated total cost of $2.8 million.

Median episodes per person (10; IQR: 5–21) and associated hospital costs ($44,412; IQR: $26,807–$70,680) were highest during 7 to 12 months after diagnosis and declined thereafter. ED costs followed a similar trend (median $1035; IQR: $0–$2886), with limited use beyond 12 months.

In an analysis of differences by LOS, total and median costs were higher for those with greater total time in hospital. However, the cost per hospital day was highest for those with shorter total LOS, suggesting more intensive or acute care within shorter hospitalizations (Table S3). Among patients who survived ≤ 6 months (approximately 50% of the cohort), the median number of hospital episodes was 4 (IQR: 2–7), and the total median length of stay was 16 days (IQR: 8–31). While not all patients exceeded a total of 28 days, many reached this threshold rapidly due to multiple admissions over a short period, underscoring the high inpatient burden and intensive healthcare needs in this group.

### 3.2 Healthcare Use and Costs in the First Year After Diagnosis, by Survival Time

**FIGURE 1:**
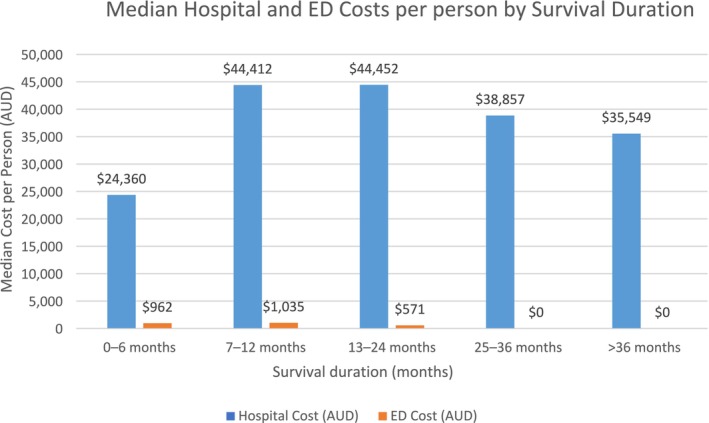
Median hospital and ED costs per person in the first 12 months following diagnosis for Queensland adults diagnosed with pancreatic cancer between 1 July 2011 and 30 June 2015 based on length of survival.

Given the substantially higher healthcare use and associated costs during the first 12 months post‐diagnosis, we examined hospital episodes, ED presentations, and associated costs in this period, based on how long individuals survived following diagnosis (Figure 1). This analysis is conditional on patients surviving for a specified duration (e.g., 0–6 months, 7–12 months, etc.), and reports the healthcare use and costs incurred within the first year of life for each survival group.

Median hospital costs per person in the first year were lowest among those who survived ≤ 6 months ($24,360; IQR $13,457–$40,967), but this group still contributed the largest share of total hospital costs during the first year (38%; $31 million), reflecting both high mortality and intensive resource use shortly after diagnosis. In contrast, patients who survived 7 to 12 months had the highest median hospital cost per person ($44,412; IQR $26,807–$70,680), followed closely by those surviving 13 to 24 months ($44,452; IQR $25,955–$66,222).

Patients who survived > 36 months had the lowest total hospital costs in the first year ($12.1 million) and among the lowest median hospital costs per person ($35,549; IQR $18,416–$54,359).

For ED presentations, the ≤ 6‐month survival group again contributed the highest total cost ($1.28 million; 46% of total ED costs), though the highest median cost per person was observed in the7 to 12 months group ($1035; IQR $0–$2886).

### 3.3 Hospital and ED Use and Costs in the First Year After Diagnosis, by Patient Group

Table 3 presents the median Hospital and ED use and associated costs to the Queensland healthcare system across different patient and treatment‐related characteristics. Those aged 76+ years had substantially lower median hospital and ED costs and fewer hospital episodes per person than those in other age groups (*p* < 0.05). First Nations Australians had greater median costs than other Australians for both hospital and ED (*p* < 0.05). Those in metropolitan areas cost the healthcare system, on average (based on median), slightly more than those in remote areas for hospital episodes but less for ED presentations (*p* < 0.05). The median hospital and ED costs per person were higher for those who lived in the most disadvantaged areas compared to those who lived in the least disadvantaged areas (*p* < 0.05).

**TABLE 3:** Median costs (in $AUD) and numbers of hospital episodes and ED presentations within patient subgroups for the first year following diagnosis for Queensland adults diagnosed with pancreatic cancer (2011–2015).

Variable	Median (IQR) cost ($)	Median (IQR) episodes (*n*)	Median (IQR) ED cost ($)	Median (IQR) ED presentations (*n*)
Overall	$31,661 (17,944–53,579)	5 (2–12)	$962 (0–1942)	1 (0–2)
Age groups				
18–50	$39,374 (27,864–60,388)*	6 (3–12)*	$971 (0–2,692)	1 (0–3)
51–65	$41,301 (25,226–60,521)	7 (3–21)	$963 (0–2,248)	1 (0–3)
66–75	$35,253 (21,107–56,171)	7 (3–18)	$962 (0–2,048)	1 (0–2)
76+	$22,390 (12,109–38,921)	3 (2–6)	$798 (0–1,733)	1 (0–2)
Sex				
Male	$32,852 (18,751–54,065)*	5 (2–13)	$963 (0–2,070)	1 (0–2)
Female	$30,314 (17,071–52,367)	5 (2–12)	$893 (0–1,924)	1 (0–2)
Indigenous status				
First Nations	$40,721 (23,786–65,109)*	5 (2–13)*	$1926 (646 3,283)*	2 (1–4)
Other Australians	$31,300 (17,836–53,309)	(2–9)	$962 (0–1,926)	1 (0–2)
Remoteness				
Metropolitan	$32,306 (17,896–51,043)*	5 (2–12)	$576 (0–1,738)*	1 (0–2)
Regional	$31,231 (18,265–54,359)	5 (3–13)	$971 (0–2,239)	1 (0–3)
Remote	$29,905 (17,588–57,243)	4 (2–9)	$962 (0–2,239)	1 (0–2)
IRSD^a^				
IRSD 1 & 2	$33,789 (18,018–54,984)*	4 (2–11)	$1302 (0–2,389)*	2 (0–3)
IRSD 3	$31,505 (17,621–55,152)	4 (2–10)	$963 (0–2,070)	1 (0–2)
IRSD 4 & 5	$31,805 (18,147–52,996)	5 (3–13)	$771 (0–1,907)	1 (0–2)
Comorbidity				
CCI^b^ = 0	$28,749 (15,786–49,937)*	4 (2–10)*	$943 (0–1,924)	1 (0–2)
CCI = 1	$32,591 (18,147–55,605)	5 (2–8)	$1035 (0–2,899)	1 (0–3)
CCI = 2+	$30,675 (24,253–58,414)	7 (4–20)	$1055 (0–3,198)	1 (0–4)
Morphology^c^				
Adenocarcinoma	$38177 (23082–59104)*	7 (3–19)*	$962 (0–2,070)	1 (0–2)
Carcinoma	$24766 (11629–50027)	3 (2–6)	$196 (0–1,267)	1 (0–1)
Neuroendocrine carcinoma	$33938 (18418–49814)	3 (2–7)	$347 (0–1,942)	1 (0–2)
Cancer site				
Head	$37,760 (22,748–59,316)*	5 (3–13)*	$971 (0–2,297)*	1 (0–3)
Body	$33,942 (19,687–51,891)	6 (3–20)	$815 (0–2,027)	1 (0–2)
Tail	$29,817 (18,797–51,891)	5 (3–11)	$962 (0–19,33)	1 (0–2)
Not specified	$22,021 (10,676–42,613)	3 (2–9)	$389 (0–1,480)	1 (0–2)
Other	$28,749 (17,938–50,944)	5 (2–17)	$661 (0–1,326)	1 (0–2)
Palliative care episode				
Yes	$38,458 (24,102–59,114)*	6 (3–14)*	$971 (0–2,356)*	1 (0–3)
No	$25,454 (12,310–46,009)	4 (2–11)	$360 (0–1,611)	1 (0–2)
Underwent tumor resection				
Yes	$48,783 (33,775–55,756)*	6 (3–20)*	$102 (0–1,769)*	1 (0–2)
No	$28,417 (15,682–48,153)	5 (2–12)	$962 (0–2,039)	1 (0–2)

^a^ Index of Relative Socio–demographic Disadvantage quintiles.

^b^ Charlson Comorbidity Index.

^c^ People with “Neoplasm and unspecified” morphology were excluded from subgroup analyses (*n* = 575, 27% of total cases).

* Statistical significance (*p*‐value < 0.05) indicates a difference for at least one of the groups.

Individuals with no comorbidities had lower median costs for hospital episodes and ED presentations than those with comorbidities (*p* < 0.05). The median number of hospital episodes and related costs varied significantly by tumour morphology (*p* < 0.05), ranging from $24,766 (carcinoma) to $38,177 (adenocarcinoma). Median hospital and ED costs were significantly higher for individuals whose pancreatic cancer was located in the head of the pancreas compared to all other sites (*p* < 0.05). Those who had been admitted for palliative care had substantially higher hospital and ED costs and more hospital episodes than those who had not (*p* < 0.05). Lastly, those who had undergone surgery had significantly higher hospital costs but lower ED costs compared to those who did not undergo surgery (*p* < 0.05).

### 3.4 Hospital and ED Use and Costs in the First Year After Diagnosis, by Surgical Status

We have also explored hospital episodes and costs for different groups by surgical status to better understand how tumour resection influences cost patterns (Table 4). Across all subgroups, there was a general pattern of higher hospital costs for those who had a tumour resection than for those who did not, except for patients aged 18 to 50 years, who had lower median costs if they had undergone tumour resection.

**TABLE 4:** Hospital episodes and costs (in $AUD) during the first year post‐diagnosis for patients who underwent surgery versus those who did not, among Queensland adults diagnosed with pancreatic cancer (2011–2015).

Variable	Those who underwent tumour resection (*n* = 382)		Those who did not undergo tumour resection (*n* = 1703)	
	Median costs per person (IQR)	Median episodes per person (IQR)	Median costs per person (IQR)	Median episodes per person (IQR)
Overall	$48,783 (33,775–55,756)	6 (3–20)	$28,417 (15,682–48,153)	5 (2–12)
Age groups				
18–50	$35,520 (23,902–48,573)*	3 (2–6)*	$43,494 (27,993–67,980)*	8 (4–19)*
51–65	$50,387 (35,422–68,845)	5 (3–21)	$38,637 (23,537–58,617)	7 (3–20)
66–75	$53,309 (36,615–74,178)	10 (4–22)	$31,311 (19,363–50,740)	6 (3–15)
76+	$50,140 (37,561–71,311)	6 (3–20)	$21,396 (11,412–34,603)	3 (2–6)
Sex				
Male	$50,972 (35,122–69,635)	6 (3–21)	$28,993 (16,855–49,844)	5 (2–12)
Female	$46,238 (29,527–65,237)	5 (3–17)	$27,676 (15,124–46,106)	4 (2–11)
Remoteness				
Metropolitan	$48,871 (35,578–66,756)	5 (2–20)	$28,964 (15,655–45,749)*	5 (2–12)*
Regional	$49,471 (31,537–67,479)	6 (3–20)	$30,377 (16,025–51,891)	5 (2–12)
Remote	$37,931 (29,731–74,796)	3 (2–7)	$26,631 (15,032–56,034)	4 (2–9)
IRSD^a^				
IRSD 1 &2	$43,042 (34,675–71,311)	7 (3–20)	$28,749 (15,672–51,891)	4 (2–10)*
IRSD 3	$46,238 (25,132–63,602)	5 (2–10)	$29,372 (15,191–52,231)	4 (2–10)
IRSD 4 & 5	$49,518 (34,306–67,479)	6 (3–21)	$28,417 (16,038–47,450)	5 (2–12)
Comorbidity				
CCI^b^ = 0	$44,951 (29,577–61,681)*	5 (2–16)*	$25,190 (13,900–43,390)*	4 (2–9)*
CCI = 1	$55,466 (41,645–79,107)	5 (4–19)	$26,849 (16,038–49,844)	4 (2–7)
CCI = 2+	$54,763 (41,496–73,620)	7 (5–21)	$30,675 (24,253–45,796)	7 (3–20)
Cancer site				
Head	$52,787 (38,800–68,995)*	6 (3–22)	$32,843 (19,908–56,251)*	5 (3–11)*
Body	$48,508 (24,430–71,311)	6 (3–20)	$32,591 (18,638–48,630)	5 (3–11)
Tail	$40,192 (26,300–58,632)	5 (2–11)	$26,739 (17,292–45,011)	5 (3–11)
Not specified	$41,689 (36,729–60,844)	5 (2–16)	$20,936 (10,078–40,980)	3 (2–8)
Other	$37,630 (20,312–61,681)	6 (2–18)	$26,156 (16,856–43,858)	5 (2–17)
Palliative care episode				
Yes	$57,538 (43,624–83,143)*	11 (4–24)*	$35,217 (23,005–56688)*	6 (3–12)*
No	$43,986 (28,004–61,099)	5 (2–14)	$20,936 (10,987–30,307)	3 (2–10)

*Note:* Variables with small cell sizes (*n* < 10), such as Indigenous status and morphology by tumor resection, are not shown to maintain confidentiality and data integrity.

^a^ Index of Relative Socio–demographic Disadvantage quartiles.

^b^ Charlson Comorbidity Index.

* Statistical significance (*p*‐value < 0.05) indicates a difference for at least one of the groups.

As was observed in the overall analysis (Table 4), patients in the oldest age group (76+ years) had the lowest median cost per person among those who did not undergo tumor resection, but this was not the case among those who had surgery, with the youngest patients (aged 18–50 years) having the lowest median costs in this group (Table 4).

Patterns in the costs of hospital episodes and ED presentations over 6 months and 3 years post‐diagnosis (see Tables S4 and S5) were generally similar to those seen in the first year (as shown in Table 3). Notably, the highest median costs over the 3‐year period were associated with patients diagnosed with adenocarcinoma, followed by those who underwent surgical resection with substantial overlap between these groups.

## 4 Discussion

In the first three years post‐diagnosis for 2082 Queensland adults diagnosed with pancreatic cancer in 2011–2015, hospital episodes cost an estimated $100.7 million (26,285 episodes), and ED presentations cost $3.6 million (4228 presentations). The median cost per person was $36,832 for hospital episodes and $963 for ED presentations. Most hospital and ED episodes occurred during the first 12 months after diagnosis, contributing the highest total and median number of episodes and total and median cost per person. Variations in median hospital episodes and associated costs were observed across subgroups (e.g., age groups, Indigenous status, socio‐economic disadvantage) and clinical characteristics (e.g., morphology, site, surgical status). Estimated total and average costs for pancreatic cancer vary across studies due to the differences in methodology and factors analyzed. A recent European systematic review reported a wide range of total costs per person, from EUR €802 to EUR €353,099, typically calculated over periods from one year post‐diagnosis to lifetime [3]. Our study identified higher costs and more hospital episodes during the initial 12 months post‐diagnosis, consistent with a previous Australian study [4]. In contrast, US studies observed higher costs during staging and surgical phases, with lower costs during the initial and continuing phases and a spike in the final three months [11]. Most hospital episodes and ED presentations occurred within the first year post‐diagnosis, with numbers decreasing over time due to high mortality rates. However, this decline may not affect median costs to the same extent, as medians are calculated based on the population alive at the start of each time period.

Our study observed notable variations in hospital and ED costs based on survival time during the first 12 months post‐diagnosis. Patients who survived ≤ 6 months had the lowest median costs but still accounted for 38% of hospital and 46% of ED costs, reflecting their larger numbers. Conversely, those who died between 7 and 12 months had the highest median costs likely due to ongoing treatment costs and increased healthcare utilization near life. To our knowledge, no studies have yet reported long‐term, survival‐specific healthcare costs, limiting direct comparison of our findings. Cost variations across studies could be influenced by factors such as cancer staging, comorbidities, complexity of medical needs, use of expensive treatments, and healthcare system differences such as the higher costs in the US compared to Australia [30, 31].

We found higher healthcare costs in the first year post‐diagnosis for First Nations Australians and residents of the most socio–economically disadvantaged areas, who often face barriers to early diagnosis and specialized care [32], leading to advanced‐stage cancer at presentation [33]. In contrast, older patients and those from remote areas had lower hospital episode costs compared to younger individuals, reflecting fewer comorbidities [34] and more intensive treatments in younger patients [35]. Limited studies have examined cost variations across socio–demographic groups, with most reporting higher costs among younger people [6, 10].

Consistent with previous studies, we found that individuals with comorbidities experienced higher numbers of hospital episodes and costs than those without comorbidities [36, 37]. Among histological subtypes, adenocarcinoma incurred the highest median inpatient costs. Tumours located in the head of the pancreas (44%) were linked to higher hospital and ED costs than those in the body or tail of the pancreas, likely due to anatomical location and the complexity of treatment. Head of the pancreas tumours often present with biliary obstruction and require more extensive surgery, most commonly the Whipple procedure [7] (pancreaticoduodenectomy). However, tumours in the tail of the pancreas are usually treated with distal pancreatectomy, which is a less complex and less resource‐intensive procedure. In our cohort, 98% of Whipple procedures were performed on patients with head of pancreas tumours, supporting the link between tumour location and higher inpatient resource use.

This study found significantly higher costs for those who had surgery than for those who did not, supporting previous findings that patients with resectable locoregional cancer cost more to the healthcare system [10, 30]. Furthermore, surgery influenced the cost distribution in some subgroups, with surgical patients incurring higher costs across all characteristics (age, comorbidities, tumour location) except for those aged 18 to 50. These results highlight the complex interplay between surgery, patient factors, and inpatient service use in pancreatic cancer, reinforcing the need for tailored strategies for effective management of these costs.

Lastly, hospital‐based palliative care episodes resulted in higher costs and episodes compared to those who had not been admitted for palliative care. However, the median cost per person for those who received hospital‐based palliative care was lower than that for patients who underwent tumor resection, consistent with previous studies [12, 38]. The general upward trend in healthcare costs over time may reflect advancements in treatments, increased use of multidisciplinary care, more aggressive options, and increasing staff costs with Australian guidelines emphasizing multidisciplinary management likely contributing to the rise in expenditures around 2015 [39].

This study has several limitations. First, the dataset lacked cancer staging information, limiting our ability to assess healthcare utilization and costs by disease severity. The analysis focused on inpatient hospital and emergency department services and did not include outpatient care, such as chemotherapy, follow‐up visits, or primary care services funded through the Medicare Benefits Schedule (MBS) or Pharmaceutical Benefits Scheme (PBS). As a result, some components of cancer‐related health care use and costs are not represented. This is particularly relevant when interpreting the higher inpatient costs among surgical patients, as the absence of outpatient data may overemphasize the cost burden associated with surgery while underrepresenting outpatient treatment costs for non‐surgical patients. As a result, total healthcare expenditure for pancreatic cancer is likely underestimated. Out‐of‐pocket costs were also not captured. The dataset only provided the month and year of hospital episode or ED presentation, making it impossible to determine whether such events occurred within 30 days of diagnosis or death. Socioeconomic status was derived from area‐level postcode data, which may not reflect individual financial circumstances. Our cost estimates were based on AR‐DRG assigned costs for public hospitals and average charges for private hospitals, excluding unbundled or patient‐specific services. Demographic‐based cost adjustments were applied only to public hospital data, due to the lack of equivalent data for private hospitals, introducing heterogeneity between public and private cost estimates.

Finally, while we used WHO classification to group malignant pancreatic tumor morphologies, administrative data lacked detailed pathology. Non‐specific morphology codes (e.g., 8000/3, 8010/3) were included in total cost analyses to avoid underestimation but excluded from subgroup comparisons, which may limit the specificity of morphological findings.

In conclusion, this study utilized linked administrative data to estimate hospital and ED costs for pancreatic cancer patients in Queensland, Australia, providing valuable population–level insights into inpatient resource use and cost patterns. While limited to inpatient data, the analysis of inpatient service use and costs by cancer and socio‐demographic characteristics contributes to identifying groups at risk of higher hospital‐based costs. Future research should incorporate outpatient, pharmaceutical, and indirect costs, examine staging and treatment pathways, and evaluate newer therapies. Understanding cost trade‐offs between inpatient and outpatient care will be critical for service planning. These findings provide a foundation for targeted interventions to reduce hospital‐related costs and improve patient outcomes.

## Author Contributions

**Shafkat Jahan:** conceptualization (equal); data curation (lead); formal analysis (lead); methodology (equal); project administration (lead); writing – original draft (lead); writing – review and editing (equal). **Daniel Lindsay:** conceptualization (equal); methodology (equal); writing – review and editing (supporting). **Abbey Diaz:** conceptualization (equal); methodology (supporting); writing – review and editing (supporting). **Ming Li:** conceptualization (equal); methodology (supporting); supervision (supporting); writing – review and editing (supporting). **Joan Cunningham:** conceptualization (equal); methodology (equal); supervision (equal); writing – review and editing (equal). **Gail Garvey:** conceptualization (equal); methodology (equal); supervision (Lead); writing – review and editing (equal).

## Ethics Statement

Ethical approval was obtained from the Townsville Hospital and Health Service Human Research Ethics Committee (HREC) (HREC/16/QTHS/11), Australian Institute of Health and Welfare (AIHW) HREC (EO2017/1/343), James Cook University HREC (H6678), and The University of Queensland HREC (2022/HE002538). Permission to waive consent was approved by Queensland Health under the Public Health Act 2005. No identifiable information was provided to the authors.

## Consent

The authors have nothing to report.

## Conflicts of Interest

The authors declare no conflicts of interest.

## Supporting information


**Table S1:** Morphological Types of Pancreatic Cancer Based on WHO 5th Edition Classification [16].

**Table S2:** Procedure codes for pancreatic surgery based on Australian Classification of Health Interventions [22].

**Table S3:** Total and average costs (in $AUD) based on the total length of hospital stays (LOS) for individuals diagnosed with pancreatic cancer during the first three years post‐diagnosis.

**Table S4:** Average costs (in AUD) and numbers of hospital episodes and ED presentations within patient subgroups for the first 6 months following diagnosis for Queensland adults diagnosed with pancreatic cancer (2011–2015).

**Table S5:** Average costs (in $AUD) and numbers of hospital episodes and ED presentations within patient subgroups for the first 3 years following diagnosis for Queensland adults diagnosed with pancreatic cancer (2011–2015).

## Data Availability

The datasets used during the current study are not publicly available due to privacy constraints associated with our ethics approval that explicitly prohibits the sharing of data.
