# Supplementary material for: Exploring the Costs of Hospital and Emergency Department Utilisation in the First Three Years After Diagnosis for Adults Diagnosed With Pancreatic Cancer in Queensland, Australia
Source: Cancer Med. 2025 Sep 4;14(17):e71193. doi: 10.1002/cam4.71193 (PMC12409639; doi:10.1002/cam4.71193)
Supplement: Supplementary file 1 — Table S1: Morphological Types of Pancreatic Cancer Based on WHO 5th Edition Classification [16]. [file CAM4-14-e71193-s001.docx]

Table S1: Morphological Types of Pancreatic Cancer Based on WHO 5th Edition Classification^16^

| Morphology Code | Classification Group |
| --- | --- |
| 8140/3, 8500/3, 8144/3, 8260/3, 8163/3, 8480/3, 8490/3, 8510/3, 8020/3, 8035/3, 8560/3 | PDAC |
| 8441/3, 8452/3, 8453/3, 8470/3, 8550/3, 8551/3, 8552/3, 8576/3, 8310/3, 8070/3, 8971/3 | non-PDAC Carcinoma |
| 8240/3, 8249/3, 8246/3, 8013/3, 8041/3, 8244/3, 8150/3, 8151/3, 8152/3, 8153/3, 8154/3, 8683/0, 8156/3 | Neuroendocrine Carcinomas |
| 8000/3, 8010/3, 8012/3, 8031/3, 8021/3, 8440/3, 8481/3, 8574/3, 8980/3 | Carcinoma (NOS), Neoplasm (NOS) |

Note: Only malignant tumours (behaviour code /3) were included. Neoplasms were excluded from Pancreactic Ductal Adenocarcinoma (PDAC)-specific subgroup comparisons but included in total cost analyses.
