# Supplementary material for: Exploring the Costs of Hospital and Emergency Department Utilisation in the First Three Years After Diagnosis for Adults Diagnosed With Pancreatic Cancer in Queensland, Australia
Source: Cancer Med. 2025 Sep 4;14(17):e71193. doi: 10.1002/cam4.71193 (PMC12409639; doi:10.1002/cam4.71193)
Supplement: Supplementary file 2 — Table S2: Procedure codes for pancreatic surgery based on Australian Classification of Health Interventions [22]. [file CAM4-14-e71193-s002.docx]

S2 Procedure codes for pancreatic surgery based on Australian Classification of Health Interventions.^22^

| ACHI Code | Descriptor |
| --- | --- |
| 3058400 | Pancreaticoduodenectomy with formation of stoma/Whipple procedure |
| 3057700 | Major pancreatic or retro pancreatic dissection |
| 3057800 | Excision of lesion of pancreas or pancreatic duct |
| 3058600 | Anastomosis of pancreas to duodenum |
| 3058300 | Distal pancreatectomy |
| 3058700 | Anastomosis of pancreas to Roux-en-Y loop of jejunum |
| 3058900 | Pancreatojejunostomy |
| 3059300 | Pancreatectomy |
| 3059301 | Pancreatectomy with splenectomy |
