# Supplementary material for: Exploring the Costs of Hospital and Emergency Department Utilisation in the First Three Years After Diagnosis for Adults Diagnosed With Pancreatic Cancer in Queensland, Australia
Source: Cancer Med. 2025 Sep 4;14(17):e71193. doi: 10.1002/cam4.71193 (PMC12409639; doi:10.1002/cam4.71193)
Supplement: Supplementary file 3 — Table S3: Total and average costs (in $AUD) based on the total length of hospital stays (LOS) for individuals diagnosed with pancreatic cancer during the first three years post‐diagnosis. [file CAM4-14-e71193-s003.docx]

Table S3. Total and average costs (in $AUD) based on the total length of hospital stays (LOS) for individuals diagnosed with pancreatic cancer during the first three years post-diagnosis.

| Total length of stay^a,b^ | Total cost | Total number of episodes^c^ | Median cost  per person (IQR) | Median number of episodes per person (IQR) | Median cost per day (IQR) |
| --- | --- | --- | --- | --- | --- |
| <=7 days (n=273) | $6,735,507 | 853 | $21,449  (13,093–32,741) | 3 (2–4) | $6,347  (3,711–11,503) |
| 8 days to <=28 days (n=671) | $25,246,906 | 4,644 | $31,875  (20,969–47,867) | 5 (4-9) | $1,992  (1,255–3,077) |
| >28 days (n=828) | $62,558,818 | 20,267 | $65,047  (43,633–96,252) | 18(8–34) | $1,102  (787–1,659) |

Note: Median number of LOS per episode 3 days (2–6)

1. The total length of stay (LOS) for each person is calculated by summing the LOS of all episodes. Each category

(<=7 days, 8-28 days, >28 days) represents the total LOS for individuals whose cumulative LOS falls within the respective range.

1. LOS for 641 episodes from 314 individuals were excluded, as these episodes occurred prior to the diagnosis date.
2. The total number of episodes reflects the number of hospital episodes for individuals within each category.
