# Supplementary material for: Exploring the Costs of Hospital and Emergency Department Utilisation in the First Three Years After Diagnosis for Adults Diagnosed With Pancreatic Cancer in Queensland, Australia
Source: Cancer Med. 2025 Sep 4;14(17):e71193. doi: 10.1002/cam4.71193 (PMC12409639; doi:10.1002/cam4.71193)
Supplement: Supplementary file 4 — Table S4: Average costs (in AUD) and numbers of hospital episodes and ED presentations within patient subgroups for the first 6 months following diagnosis for Queensland adults diagnosed with pancreatic cancer (2011–2015). [file CAM4-14-e71193-s005.docx]

S4. Average costs (in AUD) and numbers of hospital episodes and ED presentations within patient subgroups for the first 6 months following diagnosis for Queensland adults diagnosed with pancreatic cancer (2011–2015).

| Variable | Median (IQR) cost ($) | Median (IQR) episodes (n) | Median (IQR) ED cost ($) | Median (IQR) ED presentations (n) |
| --- | --- | --- | --- | --- |
| Overall | $24,015 (13528–41044) | 4 (2–10) | $1,381 (0–1585) | 1 (0–2) |
| Age groups  18–50  51–65  66–75  76+ | $31,623 (20,817–$47,408)*  $30,708 (1,7608–47,925)  $25,460 (15,206–44,196)  $18,355 (9,427–30,636) | 4 (2–10)*  5 (3–13)  5 (2–13)  3 (2–5) | $665 (0–2,229)*  $771(0–1,924)  $393 (0–1,556)  $506 (0–1,275) | 1 (0–2)*  1 (0–2)  1 (0–2)  1 (0–1) |
| Gender  Male  Female | $24,852 (14,617–43,007)*  $23,713 (12,343–39,281) | 4 (2–10)*  3 (2–9) | $771 (0–1,769)*  $187 (0–1,348) | 1 (0–2)*  1 (0–2) |
| Indigenous status  First Nations  Other Australians | $30,301 (14,616–53,728)  $23,922 (13,479–40,726) | 3 (2–7)  4 (2–10) | $963 (0–2,248)*  $521 (0–1,556) | 1 (0–2)*  1 (0–2) |
| Remoteness  Metropolitan  Regional  Remote | $24,227 (12,785–39,468)  $24,029 (14,515–43,510)  $23,319 (10,066–41,291) | 4 (2–10)*  4 (2–10)  3 (1–7) | $0 (0–1275)*  $962 (0–1907)  $592 (0–1586) | 0 (0–1)*  1 (0–2)  1 (0–2) |
| IRSD^a^  IRSD 1 &2  IRSD 3  IRSD 4 & 5 | $25,541 (13,802–45,375)  $24,351 (13,945–42,130)  $23,827 (13,413–39,970) | 4 (2–10)*  3 (2–8)  4 (2–10) | $971 (0–2070)*  $766 (0–1733)  $0 (0–1326) | 1 (0–3)^*^  1 (0–2)  0 (0–2) |
| Comorbidity  CCI^b^ =0  CCI=1  CCI=2+ | $22,154 (11,966–38,812)*  $24,281 (14,715–45,539)  $26,286 (6,780–39,743) | 3 (2–8)*  3 (2–6)  5 (2–18) | $409 (0–1,357)*  $963 (0–1,926)  $449 (0–1,733) | 1 (0–2)*  1 (0–2)  1 (0–2) |
| Morphology  Adenocarcinoma  Carcinoma  Neuroendocrine carcinoma | $27,988(16,497–45,712)*  $18,827 (9,259–31,640)  $25,056 (14,502–31,640) | 5 (3–13)*  2 (2–5)  2 (1–4) | $521 (0–1,733)*  $0 (0–963)  $0 (0–971) | 1 (0–2)*  0 (0–1)  0 (0–1) |
| Cancer site  Head  Body  Tail  Not specified  Other | $28,986 (17,232–46,372)*  $21,856 (13,747–36,565)  $24,388 (14,656–37,629)  $17,399 (7,984–32,651)  $22,021 (13,747–36,686) | 4 (2–10)*  5 (2–14)  4 (2–9)  3 (1–7)  4 (2–12) | $962 (0–1,868)*  $0 (0–1,424)  $893 (0–1,769)  $0 (0–1,267)  $0 (0–1,035) | 1 (0–2)*  0 (0–2)  1 (0–2)  0 (0–1)  0 (0–1) |
| Palliative care episode  Yes  No | $28,971 (16,985–46,070)*  $20,095 (10,987–36,615) | 5 (3–11)*  3 (2–9) | $962 (0–1,924)*  $0 (0–1,035) | 1 (0–2)*  0 (0–1) |
| Underwent tumour resection  Yes  No | $39,286 (25,446–53,503)*  $21,517 (11,842–35,712) | 4 (2–12)*  4 (2–9) | $0 (0–971)*  $771 (0–1,733) | 0 (0–1)  1(0–2) |

* Statistical significance (p–value <0.05) indicates a difference for at least one of the groups.

a. Index of Relative Socio–demographic Disadvantage quartiles

b. Charlson Comorbidity Index

c. People with "Neoplasm and unspecified" morphology were excluded from subgroup analyses (n = 575, 27% of total cases).
